# Supplementary material for: Structural insights into the antibacterial function of the Pseudomonas putida effector Tke5
Source: EMBO J. 2026 Jan 12;45(4):1229–44. doi: 10.1038/s44318-025-00689-6 (PMC12909789; doi:10.1038/s44318-025-00689-6)
Supplement: Supplementary file 1 — Appendix [file 44318_2025_689_MOESM1_ESM.pdf]

Appendix for:

Structural insights into the antibacterial function of the *Pseudomonas putida* effector Tke5

Table of Contents

Item **Page**

Appendix Figure S1. Cryo-EM data processing workflow for the Pseudomonas putida Tke5 toxin. 2

Appendix Figure S2. Cryo-EM maps and data quality 3

Appendix Figure S3. Tap3-Tke5 Map-to-model fits. 6

Appendix Figure S4. Secondary structure diagram of Tap3 (290 modelled residues). 7

Appendix Figure S5. Secondary structure diagram of MIX (222 modelled residues). 8

Appendix Figure S6. Secondary structure diagram of the α+ β-domain. 10

Appendix Figure S7. All hydrogen bonds between Tap3 and Tke5. 11

Appendix Figure S8. The structural fold of the Tke5 MIX domain. 13

Appendix Figure S9. Hydrogen bonds between Tke5 domains. 14

Appendix Figure S10. Transmembrane helices predicted for Tke5 using TMHMM v2.0 based on a Hidden Markov Model. 15

Appendix Figure S11. Sequence alignments of PpTap3 and PaTap6 and *Pp*Tke5, *Pa*Ptx2, and *Bf*Bte2. 16

Appendix Figure S12. Structural comparison of Tke5 and Ptx2 domains. 18

Appendix Figure S13. AF3 model confidence for the predicted pore state of Tke5608-996. 20

APPENDIX TABLES ***22***

Appendix Table S1. Salt-bridge and hydrogen bond interactions between Tap3 and Tke5. Interactions were computed in PDBsum. 22

Appendix Table S2. Salt-bridge and hydrogen bond interactions between Tke5 MIX domain and α+β domain. 23

Appendix Table S3. Table showing the 34 α-helices of the α-region of Tke5. 24

Appendix References ***26***

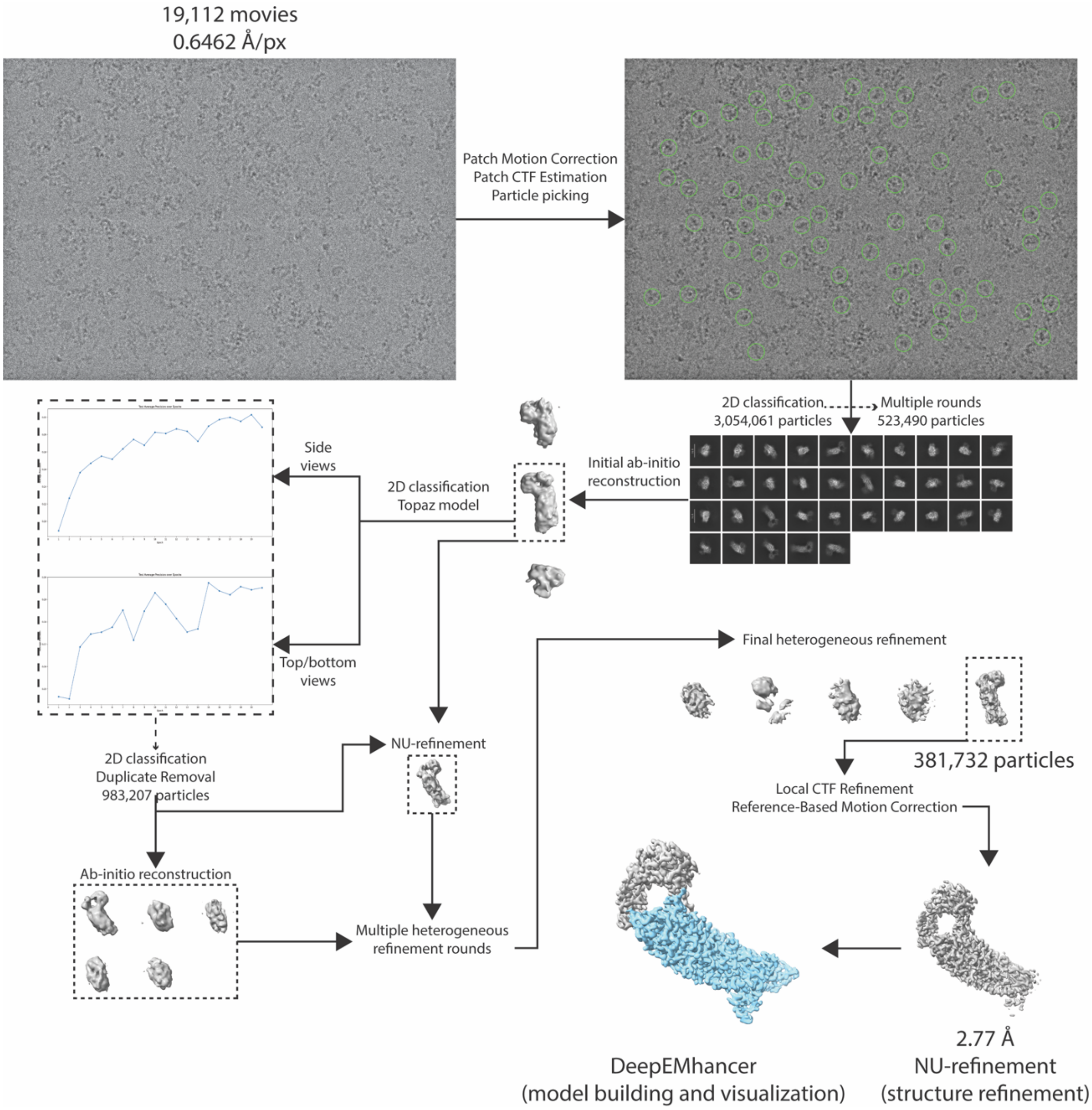

Appendix Figure S1. Cryo-EM data processing workflow for the Pseudomonas putida Tke5 toxin. Workflow encompassing data acquisition through to the reconstruction of the final 2.8 Å resolution map. Cryo-EM movies were processed in CryoSPARC v.4.5.3(Punjani *et al*, 2017). Initial particle picking was refined through 2D classification iterations, followed by ab initio reconstruction and selection of the best volume. Focused picking with Topaz(Bepler *et al*, 2019), along with further 2D classifications, improved both particle quantity and quality. Five new ab initio volumes were generated using the new particles, and the best volume from the previous ab initio was refined with the updated particle set. All six volumes were included in subsequent heterogeneous refinement cycles. The best class from the final round of heterogeneous refinement was selected for post-processing. The fully solved density map was further enhanced using DeepEMhancer(Sanchez-Garcia *et al*, 2021).

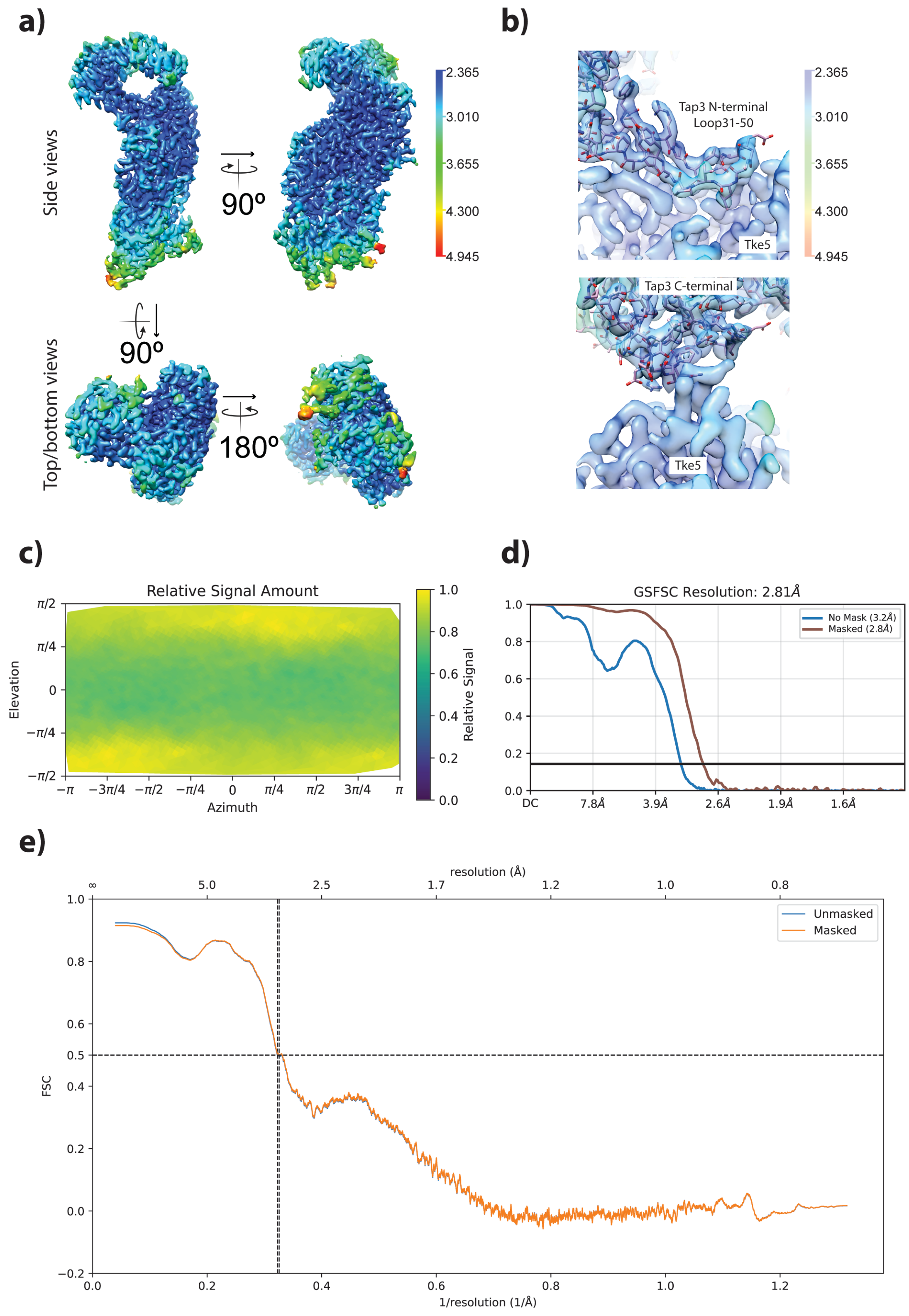

Appendix Figure S2. Cryo-EM maps and data quality. **(a)** Local resolution of the Tap3-Tke5 EM map, represented in the surface as a gradient with UCSF Chimera(Pettersen *et al*, 2004). Different side and top/bottom views are shown. Resolution estimation was based on a Fourier Shell Correlation (FSC) threshold of 0.5. **(b)** Zoomed local resolution EM map in the N-terminal Loop$^{31\text{-}50}$ and the C-terminal of Tap3 residues (atomic stick representation) interacting with Tke5 residues (no structure is shown, only the cryo-EM

map). **(c)** Relative signal amount plot of the EM map. CryoSPARC Orientation Diagnostics was used to calculate the Sampling Compensation Factor (SCF*) and the corrected Fourier Amplitude Ratio (cFAR). SCF* and cFAR values of 0.827 and 0.57 indicate no orientation bias or anisotropy. **(d)** Global resolution estimation of the EM map. Gold-standard Fourier shell correlation (GSFSC) curves were calculated with and without masking. The masked resolution is estimated at 2.8 Å using a 0.143 cutoff criterion. **(e)** Map-to-model FSC between the refined atomic model and the cryo-EM map computed in Phenix(Afonine *et al*, 2018). The FSC drops below 0.5 at 3.1 Å, indicating the resolution at which the model reliably fits the map. e) Map-to-model FSC between the refined atomic model and the cryo-EM map computed in Phenix(Afonine *et al*, 2018). The FSC drops below 0.5 at 3.1 Å, indicating the resolution at which the model reliably fits the map.

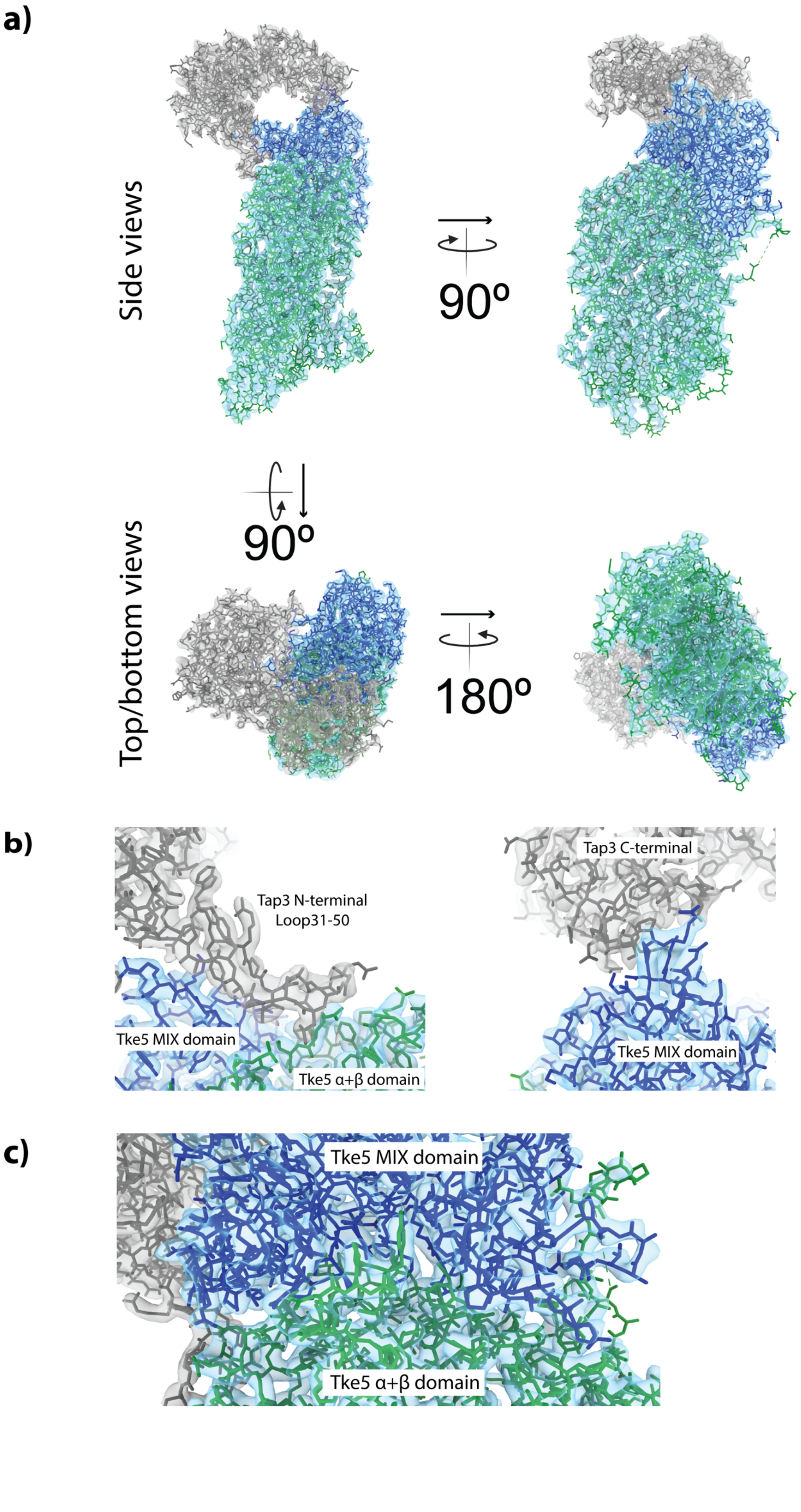
a)
Side views
90º
90º
Top/bottom views
180º
b)
Tap3 N-terminal
Loop31-50
Tke5 MIX domain
Tke5 α+β domain
Tap3 C-terminal
Tke5 MIX domain
c)
Tke5 MIX domain
Tke5 α+β domain

Appendix Figure S3. Tap3-Tke5 Map-to-model fits.

Map-to-model fit of (**a**) side and top/bottom view of Tap3–Tke5 complex, **(b)** zoomed views of interaction site residues between Tap3 N- and C-terminal and Tke5, and **(c)** detailed view of the interface between MIX domain and α+β domain of Tke5. All views were generated in ChimeraX.

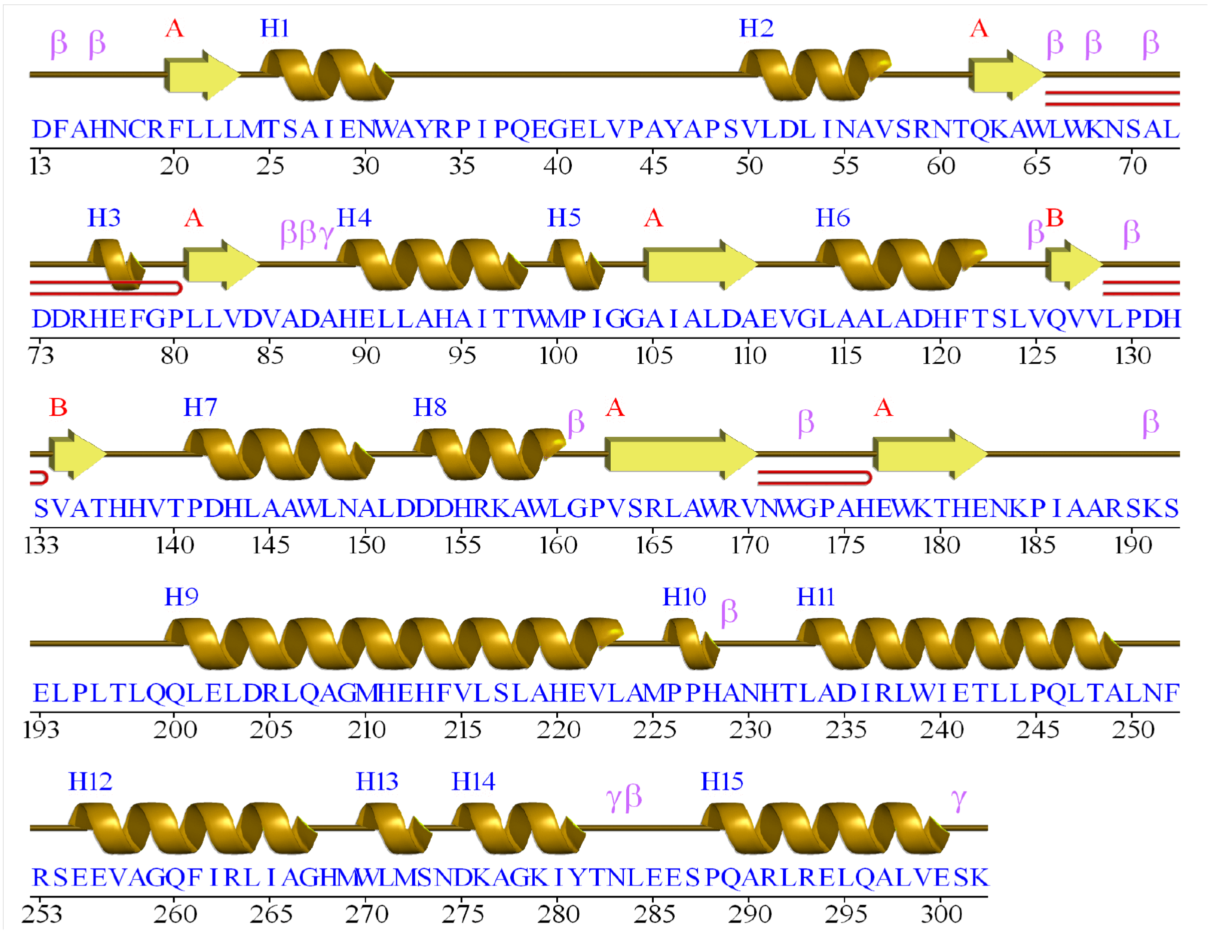

Appendix Figure S4. Secondary structure diagram of Tap3 (290 modelled residues).
The diagram was generated using PDBsum(Laskowski *et al*, 2018). It displays the secondary structure elements, including 15 α-helices, in gold, and 2 β-sheets (A, with 7 β-strands; and B, with 2 β-strands), shown in yellow. Additionally, the diagram highlights β-turns (14), γ-turns (3), and β- hairpins (3).

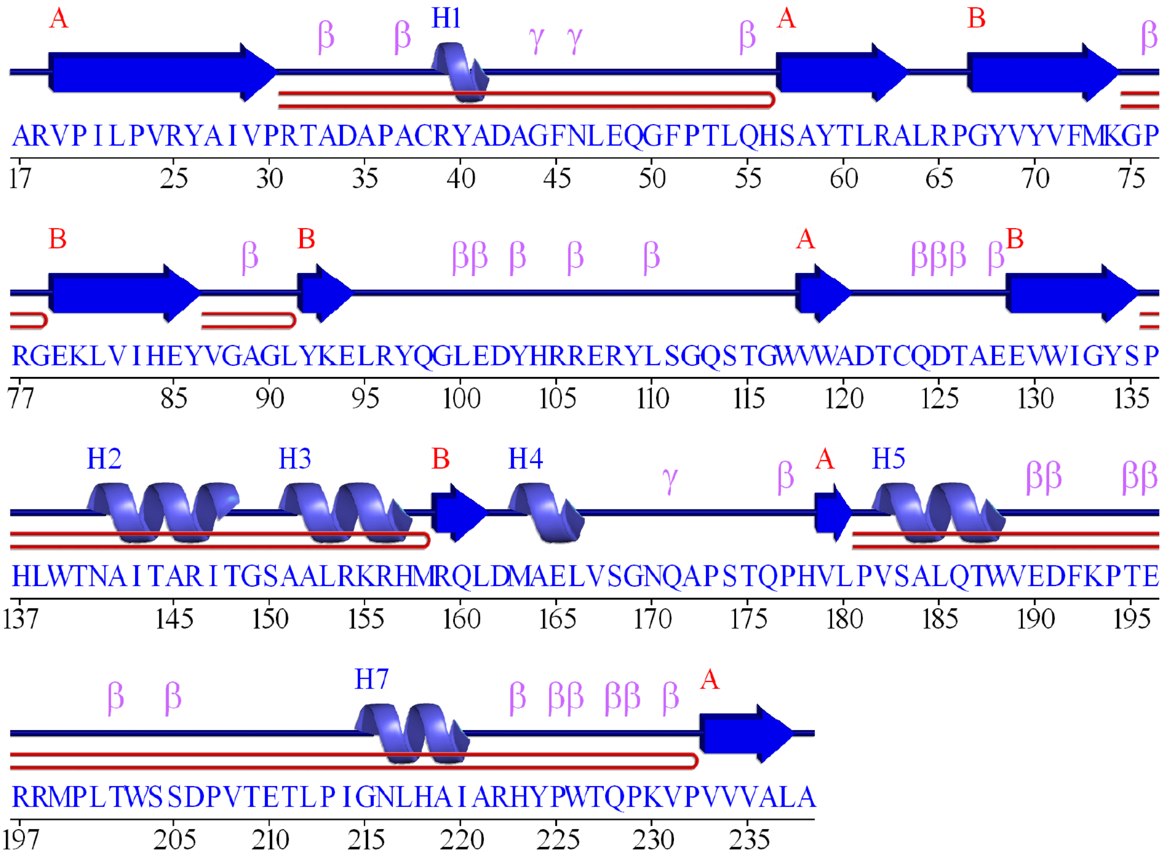

Appendix Figure S5. Secondary structure diagram of MIX (222 modelled residues).
The diagram was generated using PDBsum. It displays the secondary structure elements, including 7 α-helices, in light blue, and 2 β-sheets (A, with 5 β-strands; and B, with 5 β-strands), shown in dark blue. Additionally, the diagram highlights β-turns (27), γ-turns (3), and β-hairpins (5).

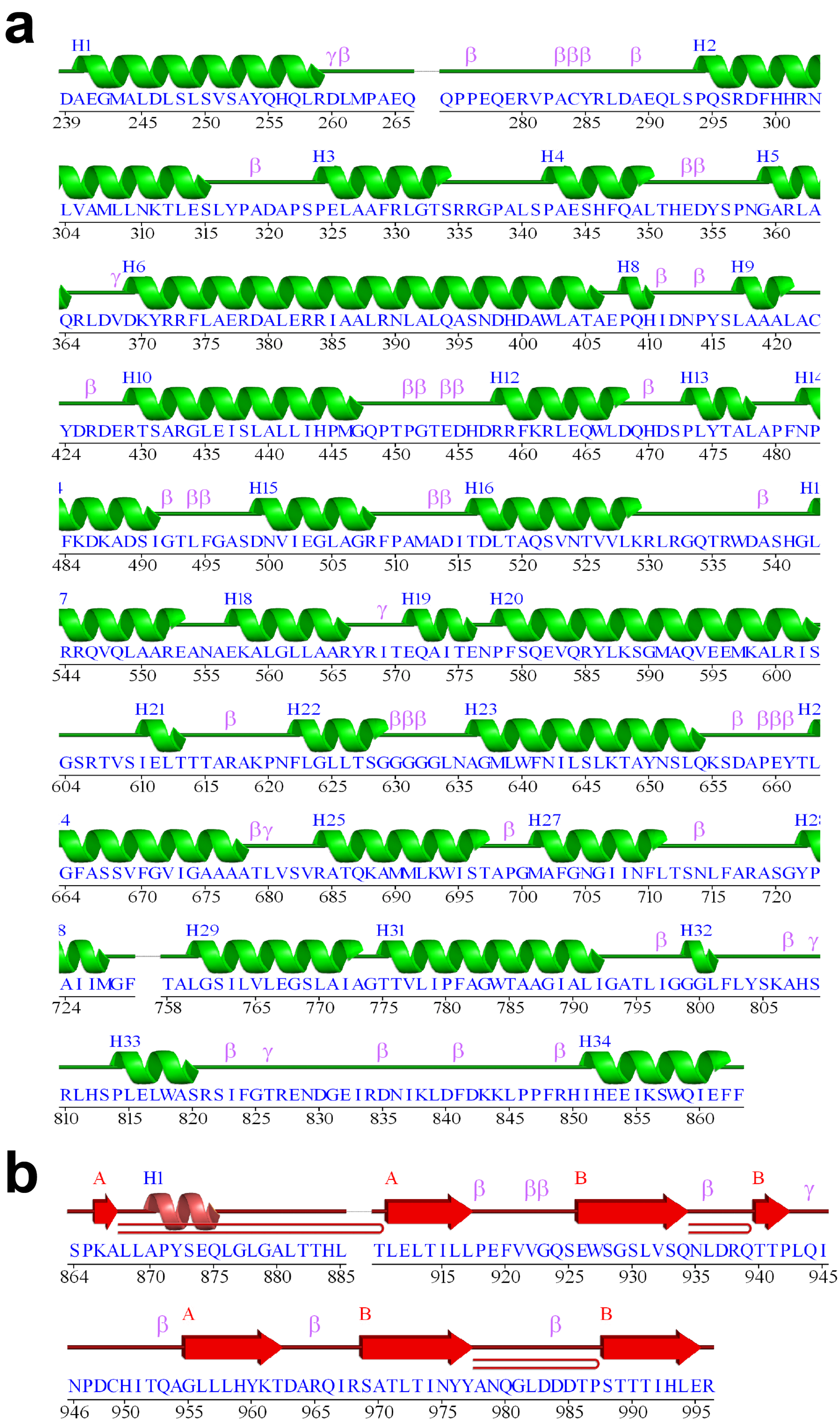
a
DAEGMALDLSLSVSAYQHQLRDLMPAEQ
QPPEQERVPACYRLDAEQLSPQSRDFHHRN
LVAMLLNKTLESLYPADAPSPELAAFRLGTSRRGPALSPAESHFQALTHEDYSPNGARLA
QRLDVDKYRRFLAERDALERRIAALRNLALQASNDHDAWLATAEPQHIDNPYSLAAALAC
YDRDERTSARGLEISLALLIHPMGQPTPGTEDHDRRFKRLEQWLDQHDSPLYTALAPFNP
FKDKADSIGTLFGASDNVIEGLAGRFPAMADITDLTAQSVNTVVLKRLRGQTRWDASHGL
RRQVQLAAREANAEKALGLLAARYRITEQAITENPFSQEVQRYLKSGMAQVEEMKALRIS
GSRTVSIELTTTARAKPNFLGLLTSGGGGGLNAGMLWFNILSLKTAYNSLQKSDAPEYTL
GFASSVFGVIGAAAATLVSVRATQKAMMLKWISTAPGMAFGNGIINFLTSNLFARASGYP
AIIMGF
TALGSILVLEGSLAIAGTTVLIPFAGWTAAGIALIGATLIGGGLFLYSKAHS
RLHSPLELWASRSIFGTRENDGEIRDNIKLDFDKKLPPFRHIHEEIKSWQIEFF
b
SPKALLAPYSEQLGLGALTTHL
TLELTILLPEFVVGQSEWSGSLVSQNLDRQTTPLQI
NPDCHITQAGLLLHYKTDARQIRSATLTINYYANQGLDDDTPSTTTIHLER

Appendix Figure S6. Secondary structure diagram of the α+ β-domain. **(a)** The α-region is shown (590 modelled residues), and **(b)** the β-rich region is shown (109 modelled residues). The diagram was generated using PDBsum. **(a)** It displays the secondary structure elements, including 34 α-helices, shown in green. Additionally, the diagram highlights β-turns (40) and γ-turns (7). See Appendix Table 3 for more details, including predicted TM helices. **(b)** It displays the secondary structure elements, including 1 α-helices, in light red, and 2 β-sheets (A, with 3 β-strands; and B, with 4 β-strands), shown in dark red. Additionally, the diagram highlights β-turns (7), γ-turns (1), and β-hairpins (3).

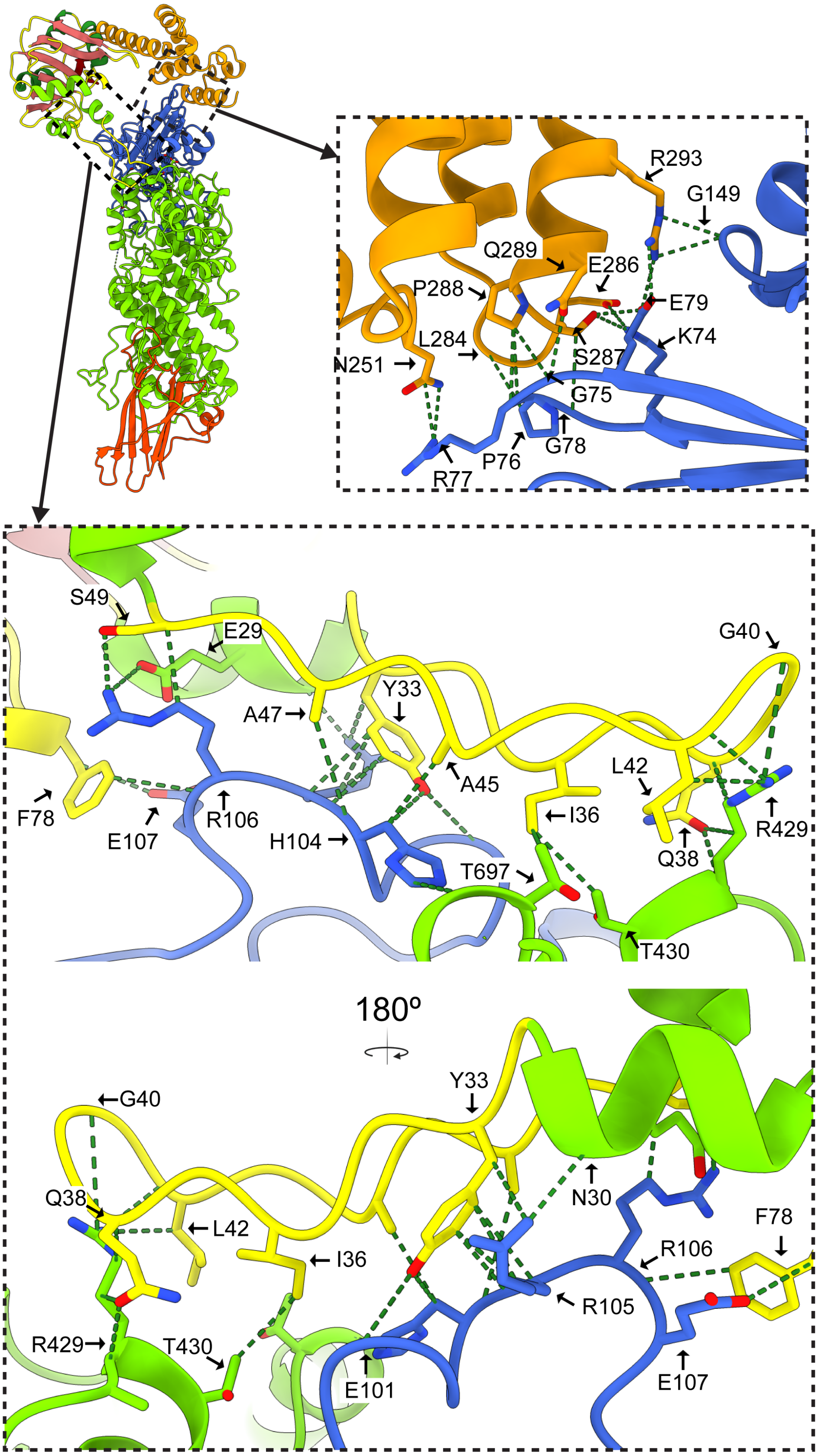

Appendix Figure S7. All hydrogen bonds between Tap3 and Tke5. An overview of the structure is shown using the same colour scheme as in Fig. 3, with quadrants indicated for zoomed-in views. The upper quadrant highlights the interaction between the α-helical bundle of Tap3 (in orange) and the MIX domain (in blue). The lower quadrant focuses on the interaction between the Tap3-Loop$^{31\text{-}50}$ (in yellow) and both the MIX domain and the α-domain (in green), shown with a 180° rotation to reveal all hydrogen bond interactions. Interactions were visualised

in ChimeraX(Pettersen *et al*, 2021). For a detailed list of these interactions, see Appendix Table 1.

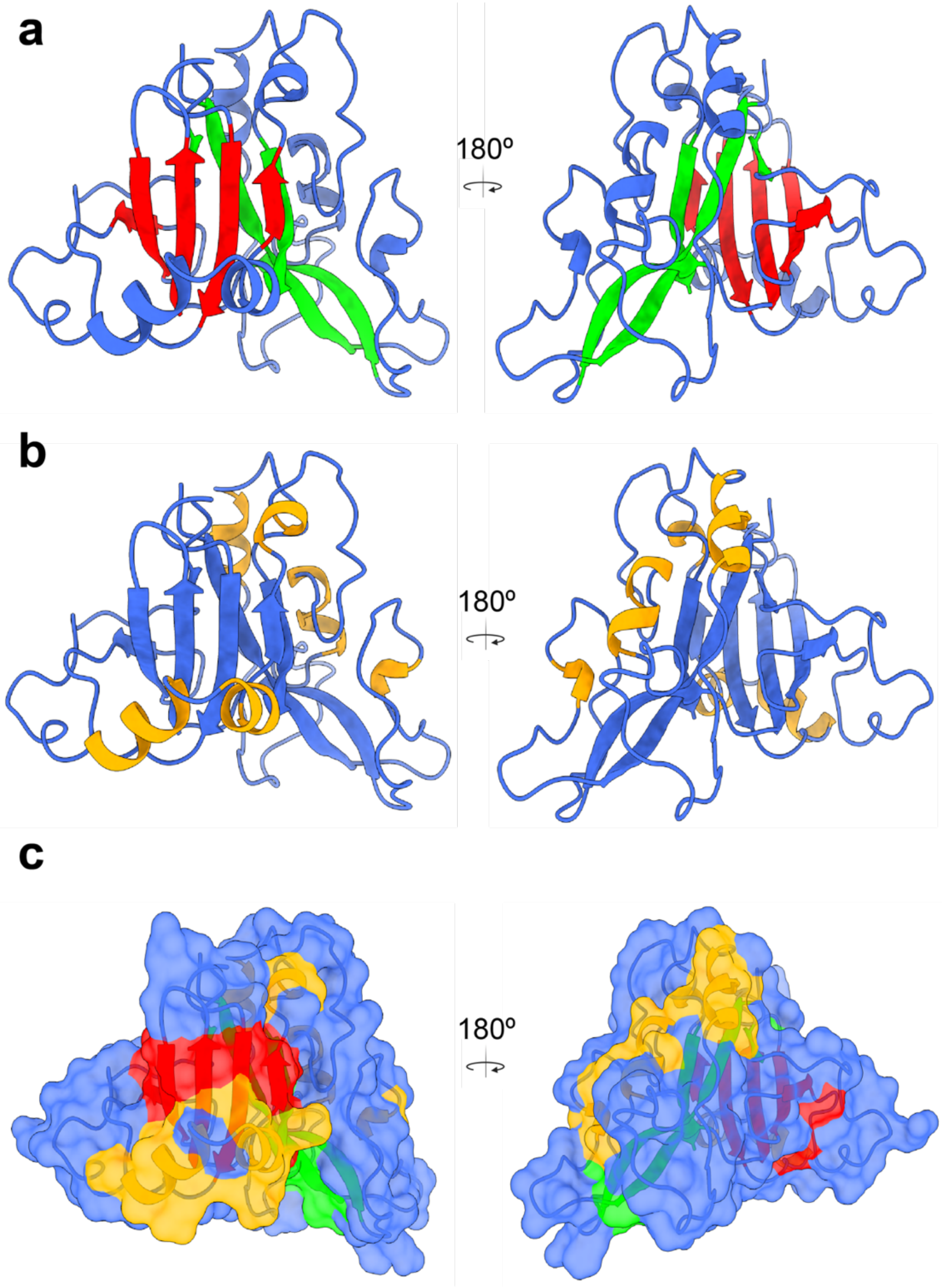

Appendix Figure S8. The structural fold of the Tke5 MIX domain. Two views of the MIX domain (residues 1-238) are shown, rotated 180º for optimal visualisation. **(a)** The two central β-sheets are distinctly highlighted, with one β-sheet coloured in red and the other coloured in green, both composed of five β-strands. **(b)** The six α-helices are displayed in orange, showing their spatial arrangement relative to the central β-sheets. **(c)** The secondary structural elements maintain the same colour scheme as in panels (a) and (b), with the addition of the MIX domain's molecular surface depicted in blue. This surface representation clearly shows the characteristic pyramid-like architecture formed by the interplay of the β-sheets and α-helices within the MIX fold.

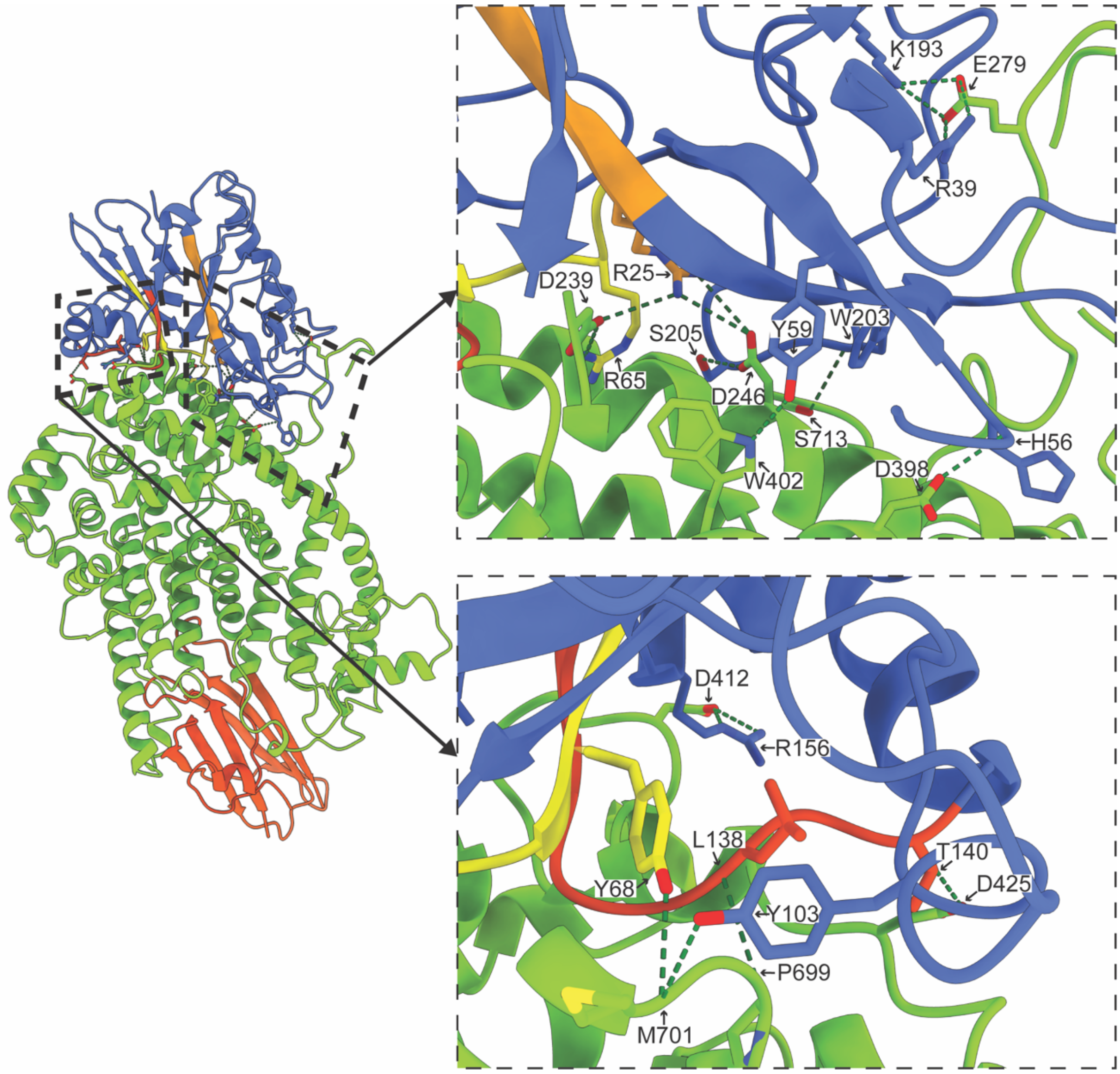

Appendix Figure S9. Hydrogen bonds between Tke5 domains. The MIX domain (shown in blue; MIX motif highlighted in red, yellow, and orange) and the α+β domain (shown in green) are displayed. For a detailed list of these interactions, see Appendix Table 2.

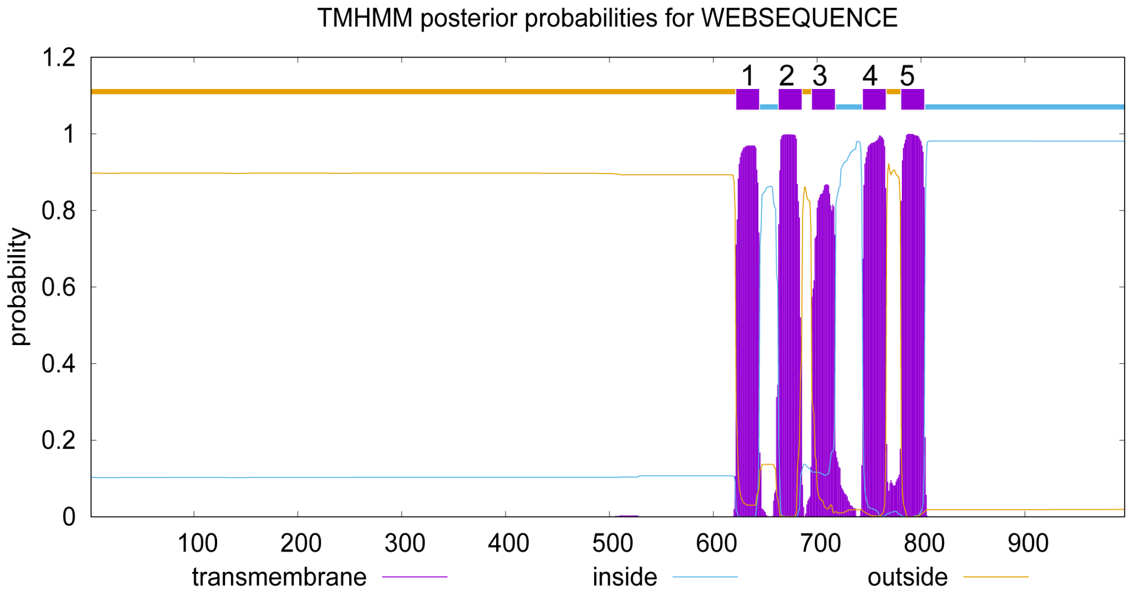

TMH1: 622-644 TMH2: 663-685 TMH3: 695-717 TMH4: 744-766 TMH5: 781-803

Appendix Figure S10. Transmembrane helices predicted for Tke5 using TMHMM v2.0 based on a Hidden Markov Model. This plot displays posterior probabilities for each amino acid residue being located in a transmembrane helix (TM helix), on the inside (cytoplasmic side), or the outside (non-cytoplasmic side) of the membrane. The predicted topology generated via the TMHMM server(Krogh *et al*, 2001) is as follows: outside (residues 1-621), TMH1 (622-644), inside (645-662), TMH2 (663-685), outside (686-694), TMH3 (695-717), inside (718-743), TMH4 (744-766), outside (767-780), TMH5 (781-803), and inside (804-996).

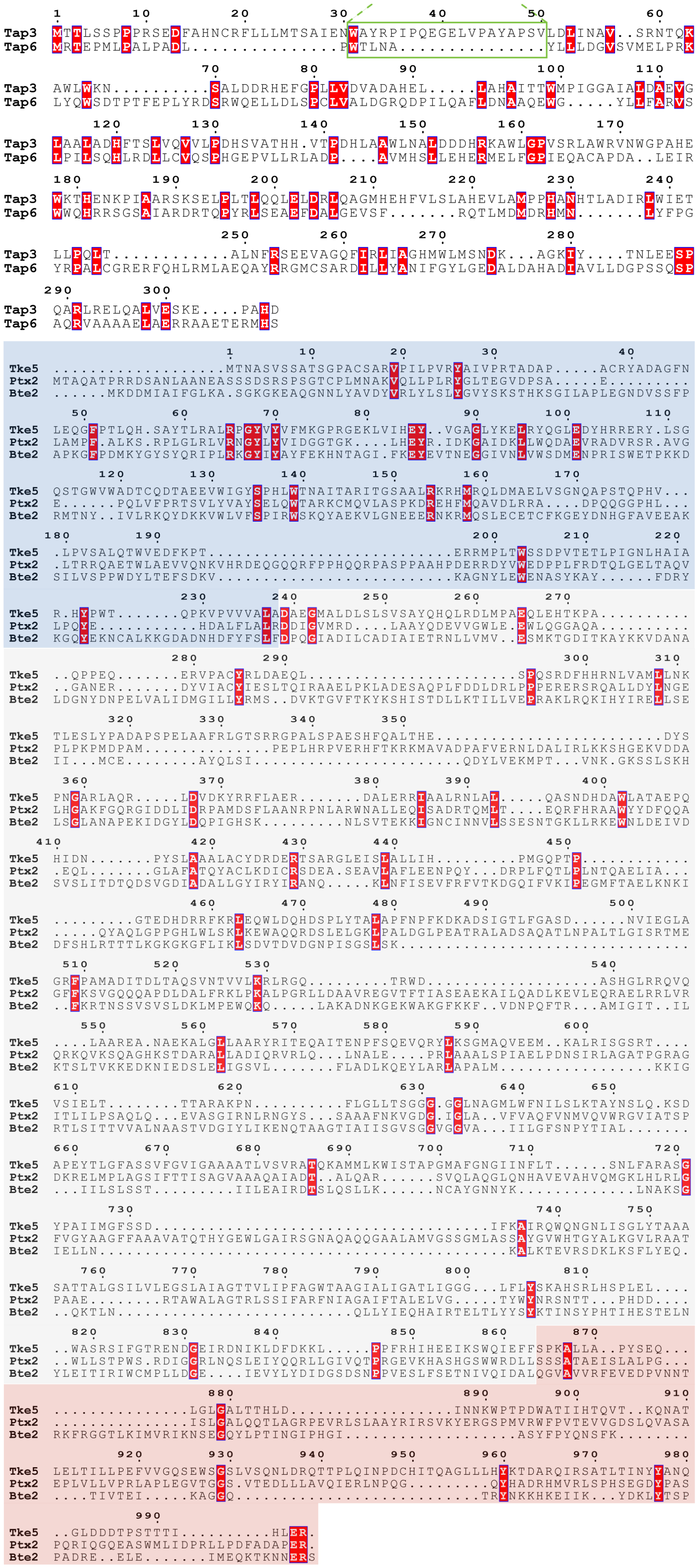

Appendix Figure S11. Sequence alignments of PpTap3 and PaTap6 and *Pp*Tke5,

*Pa*Ptx2, and *Bf*Bte2. *Pp*Tap3 and *Pa*Tap6 sequence identity shared by the aligned sequences is 16 % (top alignment). *Pp*Tke5, *Pa*Ptx2, and *Bf*Bte2 overall sequence identity among the aligned sequences is 4.05 %. In pairwise comparisons, sequence identities are 16.14 % for Tke5 and Ptx2, 13.93 % for Tke5 and Bte2, and 13.57 % for Ptx2 and Bte2. In the alignment, the MIX domain is shaded in blue, the α-domain in grey, and the β-domain in red. The alignments were generated using Clustal Omega(Madeira *et al*, 2024) and visualised with ESPript 3.0(Robert & Gouet, 2014). The "%Strict similarity" colouring scheme was applied to highlight conserved residues based on physicochemical properties.

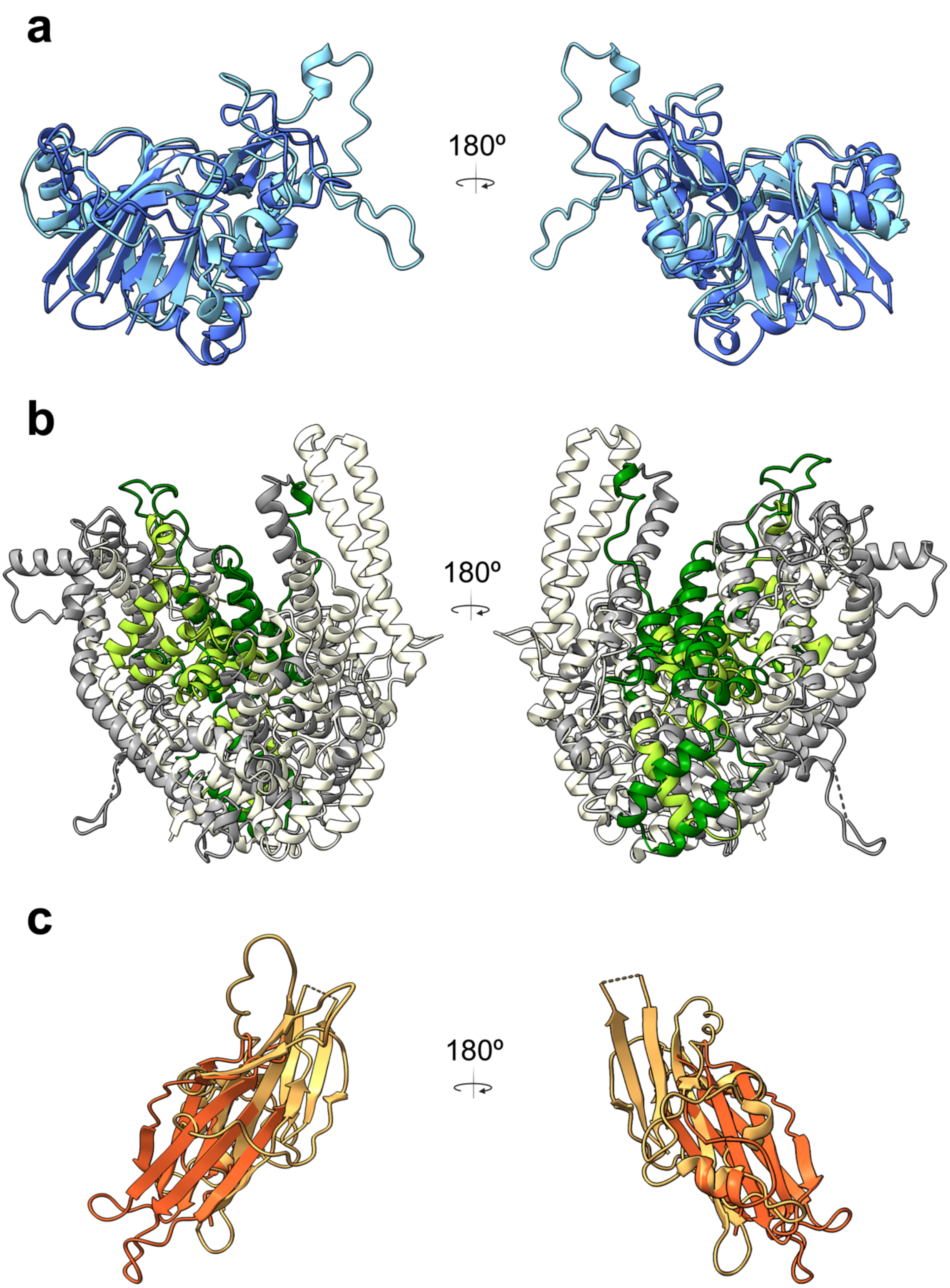

Appendix Figure S12. Structural comparison of Tke5 and Ptx2 domains. Structural alignments are shown for the MIX domain, and α- and β-regions of Tke5 and Ptx2. Two orientations of each domain are presented, rotated by 180° to facilitate visualization. Structural similarity was quantified by the root-mean-square deviation (RMSD) of Cα atom pairs, reported in Å. **(a)** MIX domain: Tke5 (residues 1–238) is shown in dark blue and Ptx2 (residues 1–258) in light blue. The RMSD calculated for 58 pruned atom pairs (conserved core) is 1.3 Å, while the RMSD over all 195 aligned atom pairs is 6.2 Å. **(b)** α-region: Tke5 (residues 239–863) is depicted in dark grey and Ptx2 (residues 259–999) in light grey. The RMSD between 15 pruned atom pairs is 0.9 Å; the RMSD across all 457 aligned pairs is 22.5 Å. Predicted transmembrane helices (TMHs)

are indicated in dark green for Tke5 (residues 608–813) and in light green for Ptx2 (residues 817–962). **(c)** β-region: Tke5 (residues 864–996) is represented in red and Ptx2 (residues 1000–1174) in orange. The RMSD between 7 pruned atom pairs is 1.3 Å; the RMSD calculated across all 87 aligned pairs is 21.9 Å.

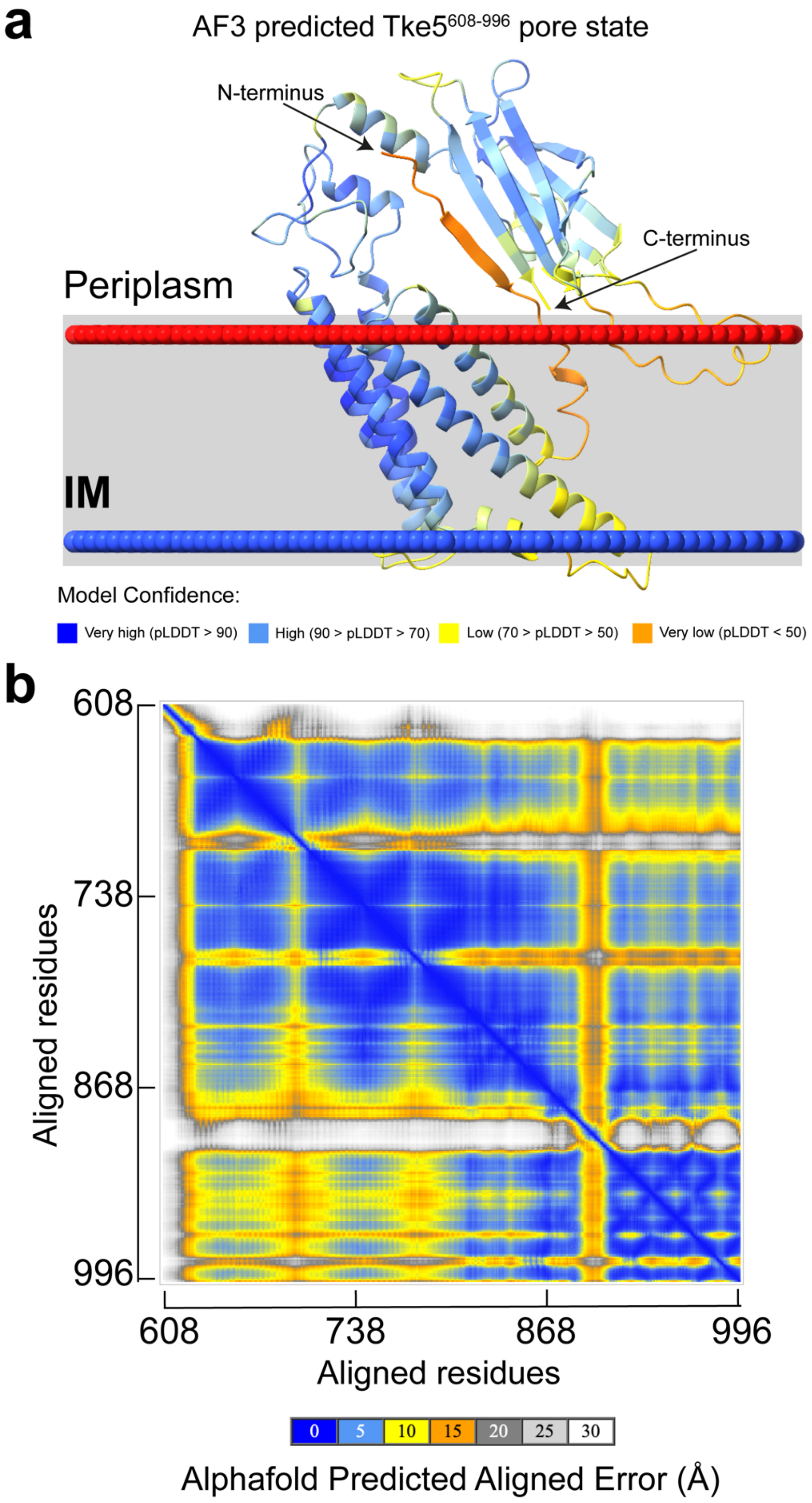

Appendix Figure S13. AF3 model confidence for the predicted pore state of Tke5608-996. **(a)** The AF3-predicted structure of Tke5$^{608\text{-}996}$ is inserted into a gram-negative inner membrane model, as suggested by PPM3.0 based on a membrane orientation prediction. The structure is color-coded according to the predicted Local Distance Difference Test (pLDDT) score, a metric indicating the confidence in the predicted atomic positions. The color scale is as follows: blue indicates very high confidence (pLDDT > 90); light blue, high confidence (90 ≥ pLDDT

> 70); yellow, moderate confidence (70 ≥ pLDDT > 50); and orange, low confidence (pLDDT ≤ 50). **(b)** Predicted Aligned Error (PAE) plot for Tke5$^{608\text{-}996}$ showing the expected positional error in angstroms for each pair of residues. Dark blue indicates very low predicted error (< 5 Å) and high confidence; yellow indicates moderate confidence with predicted error < 10 Å; orange represents low confidence with predicted error < 15 Å; gray indicates higher error and lower confidence; and white corresponds to very high predicted error (> 30 Å). The reliability of this *in silico* model as evaluated using the per-residue confidence score (pLDDT) and the Predicted Aligned Error (PAE) plot reveals that a significant portion of the model is predicted with high to very high confidence. Specifically, the regions corresponding to the putative transmembrane helices (residues 630 to 870) and the C-terminal immunoglobulin-like (Ig-like) fold (residues 911 to 996) exhibit pLDDT scores largely between 70 and 90, with many exceeding 90. The corresponding PAE plot for these domains shows low expected positional error, with values between 0-5Å, indicating high confidence in the predicted packing and relative orientation of these core structural elements. In contrast, the N-terminal residues (608 to 629), predicted to form a strand and a short helix, along with a loop region protruding from the Ig-like fold, are assigned very low confidence scores (pLDDT < 50). Such low pLDDT values are often indicative of intrinsically disordered or highly flexible regions that do not adopt a single, stable conformation.

APPENDIX TABLES

Appendix Table S1. Salt-bridge and hydrogen bond interactions between Tap3 and Tke5. Interactions were computed in PDBsum.

Tke5	Tap3	Distance (Å)
Salt-bridge interactions		
LYS74	GLU286	2.93
GLU79	ARG293	3.04
ARG106	GLU29	2.91
ARG106	GLU77	3.45
Hydrogen bond interactions		
LYS74	GLU286	2.93
LYS74	SER287	2.99
GLU79	ARG293	3.06
GLU79	SER287	2.76
GLU79	ARG293	3.04
GLU101	TYR33	2.76
ARG105	ASN30	3.13
ARG106	GLU29	2.91
ARG429	GLN38	2.94
ARG429	GLY40	3.07

Appendix Table S2. Salt-bridge and hydrogen bond interactions between Tke5 MIX domain and α+β domain. Interactions were computed in PDBsum.

MIX domain	α+β domain	Distance (Å)
Salt-bridge interactions		
ARG25	ASP239	3.80
ARG39	GLU279	3.39
ARG65	ASP239	2.83
ARG156	ASP412	3.30
ARG193	GLU279	3.06
Hydrogen bond interactions		
HIS56	ASP398	2.86
TYR59	TRP402	2.80
ARG65	ASP239	2.83
TYR68	MET701	3.10
TYR103	MET701	3.10
LEU138	PRO699	3.02
THR140	ASP425	2.78
ARG156	ASP412	3.30
LYS193	GLU279	3.06
TRP203	SER713	2.74
SER205	ASP246	3.35
SER205	ASP246	2.75

Appendix Table S3. Table showing the 34 α-helices of the α-region of Tke5. № (number), S (start), E (end), T (Type), R (number of residue), L (length), Ur (unit rise), RpT (residues per turn), P (pitch), D (deviation from ideal), S (sequence). Predicted TM Helices on the surface are coloured in blue, and TM Helices on the core are coloured in yellow. This table was built with PDBsum.

Nº	S	E	T	R	L	Ur	RpT	P	D	S
1.	A240	R259	H	20	29.73	1.47	3.57	5.25	10.2	AEGMALDLSLSVSAYQHQLR
2.	P294	S315	H	22	33.10	1.48	3.67	5.44	14.2	PQSRDFHHRNLVAMLLNKTLES
3.	P324	S334	H	11	16.73	1.48	3.67	5.43	6.0	PELAAFRLGTS
4.	P342	L350	H	9	13.76	1.48	3.57	5.29	7.4	PAESHFQAL
5.	G359	Q364	H	6	10.11	1.59	3.51	5.60	8.7	GARLAQ
6.	D369	L403	H	35	49.45	1.43	6.41	9.19	66.2	DKYRRFLAERDALERRIAALRNLALQASNDHDAWL
7.	A404	A406	G	3	-	-	-	-	-	ATA
8.	P408	H410	G	3	-	-	-	-	-	PQH
9.	L417	L421	H	5	8.07	1.51	3.58	5.39	21.2	LAAAL
10.	R429	I443	H	15	22.19	1.46	3.68	5.36	7.8	RTSARGLEISLALLI
11.	H444	G447	G	4	7.39	1.86	3.40	6.32	41.2	HPMG
12.	R458	D468	H	11	17.42	1.55	3.57	5.53	8.0	RRFKRLEQWLD
13.	P473	L478	H	6	9.58	1.49	3.46	5.14	4.9	PLYTAL
14.	N482	I491	H	10	15.33	1.48	3.63	5.38	1.4	NPFKDKADSI
15.	D499	R508	H	10	15.70	1.50	3.63	5.45	3.7	DNVIEGLAGR
16.	T516	K529	H	14	21.91	1.51	3.78	5.72	11.4	TDLTAQSVNTVVLK
17.	L543	E553	H	11	17.11	1.51	3.60	5.44	7.9	LRRQVQLAARE
18.	E557	R566	H	10	15.42	1.49	3.61	5.38	1.8	EKALGLLAAR
19.	E571	E576	H	6	9.50	1.52	3.53	5.35	5.4	EQAITE
20.	P578	S603	H	26	38.38	1.46	3.64	5.32	11.9	PFSQEVQRYLKSGMAQVEEMKALRIS
21.	I610	T613	H	4	6.84	1.66	3.49	5.81	40.7	IELT
22.	F622	G629	H	8	10.81	1.39	3.90	5.43	13.6	FLGLLTSG
23.	A636	Q654	H	19	28.51	1.47	3.63	5.34	7.5	AGMLWFNILSLKTAYNSLQ
24.	L663	A678	H	16	24.56	1.50	3.61	5.41	7.3	LGFASSVFGVIGAAAA
25.	R684	W694	H	11	17.03	1.53	3.57	5.47	11.8	RATQKAMMLKW
26.	I695	T697	G	3	-	-	-	-	-	IST

27.	M701	L711	H	11	17.11	1.52	3.59	5.44	7.7	MAFGNGIINFL
28.	Y722	M727	H	6	10.30	1.62	3.58	5.81	7.6	YPAIIM
29.	L760	S769	H	10	15.89	1.54	3.55	5.48	5.9	LGSILVLEGS
30.	L770	A773	G	4	6.80	1.49	3.93	5.86	41.7	LAIA
31.	T775	I792	H	18	26.91	1.46	3.69	5.37	11.2	TTVLIPFAGWTAAGIALI
32.	G799	L801	G	3	-	-	-	-	-	GGL
33.	P814	S820	H	7	10.87	1.53	3.54	5.40	12.2	PLELWAS
34.	I851	F862	H	12	19.44	1.57	3.54	5.56	10.3	IHEEIKSWQIEF

Appendix References

1. Punjani, A., Rubinstein, J. L., Fleet, D. J. & Brubaker, M. A. CryoSPARC: Algorithms for rapid unsupervised cryo-EM structure determination. *Nat Methods* 14, 290–296 (2017).
2. Bepler, T. *et al.* Positive-unlabeled convolutional neural networks for particle picking in cryo-electron micrographs. *Nat Methods* 16, 1153–1160 (2019).
3. Sanchez-Garcia, R. *et al.* DeepEMhancer: a deep learning solution for cryo-EM volume post-processing. *Commun Biol* 4, (2021).
4. Pettersen, E. F. *et al.* UCSF Chimera - A visualization system for exploratory research and analysis. *J Comput Chem* 25, 1605–1612 (2004).
5. Afonine, P. V. *et al.* New tools for the analysis and validation of cryo-EM maps and atomic models. *Acta Crystallogr D Struct Biol* 74, 814–840 (2018).
6. Laskowski, R. A., Jabłońska, J., Pravda, L., Vařeková, R. S. & Thornton, J. M. PDBsum: Structural summaries of PDB entries. *Protein Science* 27, 129–134 (2018).
7. Pettersen, E. F. *et al.* UCSF ChimeraX: Structure visualization for researchers, educators, and developers. *Protein Science* 30, 70–82 (2021).
8. Krogh, A., Larsson, B., Von Heijne, G. & Sonnhammer, E. L. L. Predicting transmembrane protein topology with a hidden Markov model: Application to complete genomes. *J Mol Biol* 305, 567–580 (2001).
9. Madeira, F. *et al.* The EMBL-EBI Job Dispatcher sequence analysis tools framework in 2024. *Nucleic Acids Res* 52, W521–W525 (2024).
10. Robert, X. & Gouet, P. Deciphering key features in protein structures with the new ENDscript server. *Nucleic Acids Res* 42, (2014).
