# Supplementary figures and images for: Structural insights into the antibacterial function of the Pseudomonas putida effector Tke5
Source: EMBO J. 2026 Jan 12;45(4):1229–44. doi: 10.1038/s44318-025-00689-6 (PMC12909789; doi:10.1038/s44318-025-00689-6)

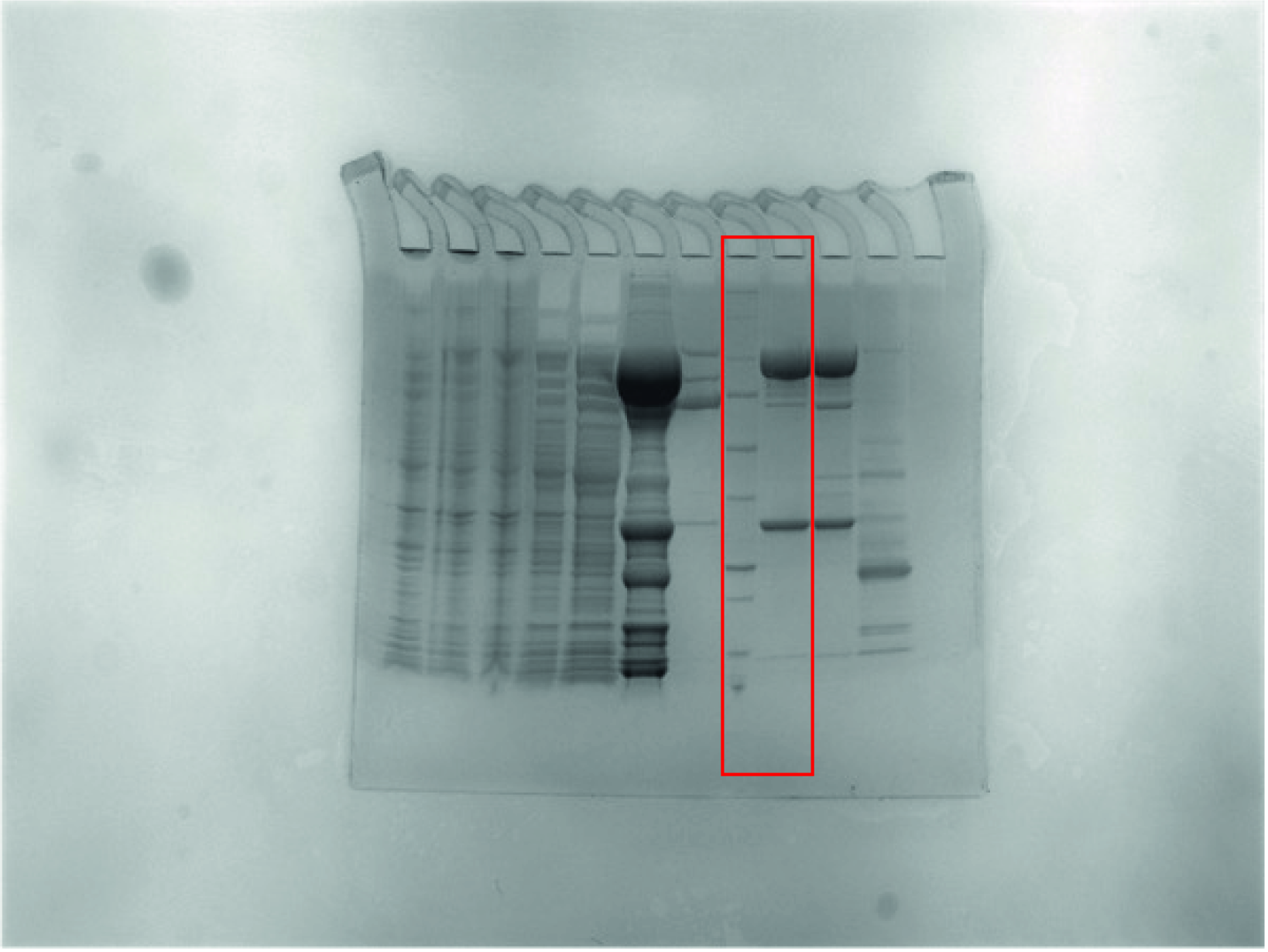

Supplement: Supplementary file 5 — Source data Fig. 1 [file 44318_2025_689_MOESM5_ESM.zip › Figure 1/1b/uncropped SDS-PAGE image.tif]

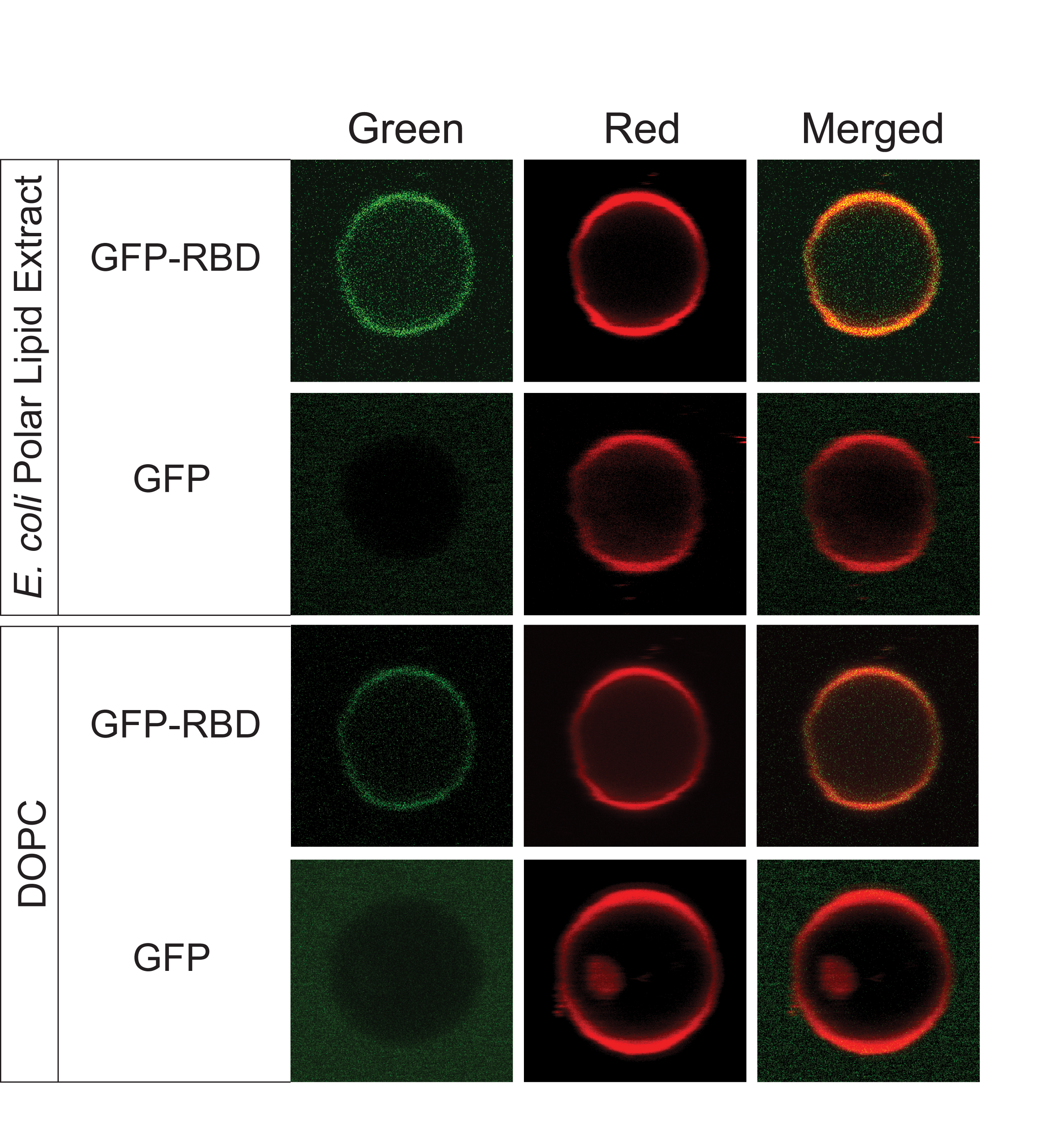

Supplement: Supplementary file 7 — Source data Fig. 6 [file 44318_2025_689_MOESM7_ESM.zip › Figure 6/6b/6b.tif]
